# A Comparative Study Assessing the Incidence and Degree of Hyperkalemia in Patients on Unfractionated Heparin versus Low-Molecular Weight Heparin

**DOI:** 10.2147/CPAA.S487288

**Published:** 2024-12-11

**Authors:** Lina Naseralallah, Dima Nasrallah, Somaya Koraysh, Shimaa Aboelbaha, Tarteel Ali Hussain

**Affiliations:** 1 Pharmacy Department, Hamad Medical Corporation, Doha, Qatar; 2 College of Medicine, QU Health, Qatar University, Doha, Qatar; 3 College of Pharmacy, QU Health, Qatar University, Doha, Qatar

**Keywords:** unfractionated heparin, low molecular weight heparin, hyperkalemia, adverse drug reactions, risk factors

## Abstract

**Background:**

Heparin and its derivates, including unfractionated heparin (UFH) and low molecular weight heparin (LMWH), are among the most commonly used anticoagulants. Nonetheless, their use has been associated with hyperkalemia.

**Objective:**

To determine and compare the incidence, magnitude, and potential risk factors of hyperkalemia in patients receiving UFH versus LMWH in a real-world clinical setting.

**Methods:**

A retrospective observational study was conducted involving all adult hospitalized patients who received UFH, dalteparin or enoxaparin. Electronic medical records were reviewed over a 12-month period, collecting data on demographic, laboratory, comorbidity, and medication-related variables. Data were analyzed using multivariate logistic regression.

**Results:**

A total of 929 patients met the eligibility criteria, with a mean age of over 40 years across all groups. Of these, 56.3%, 17.2%, and 15.7% experienced hyperkalemia with UFH, dalteparin and enoxaparin, respectively. The incidence of hyperkalemia was significantly higher with UFH compared to enoxaparin and dalteparin (p<0.001). Diabetes mellitus was associated with a higher incidence of hyperkalemia (OR 1.79, 95% CI 1.241–2.581, p=0.002), as was the concomitant use of co-trimoxazole (OR 2.244, 95% CI 1.137–4.426, p=0.02). Whilst chronic kidney disease and the use of two or more hyperkalemia-inducing agents were not statistically significant, they were retained in the model as they were associated with more than a 10% increase in the odds of hyperkalemia.

**Conclusion:**

Heparin (UFH, LMWH) administration was associated with a risk of hyperkalemia particularly in patients with diabetes mellitus and those concurrently receiving co-trimoxazole.

## Introduction

Patient safety is a core priority in modern healthcare systems as it is pivotal to providing high-quality care to patients. Multiple types of events could occur during patient care and could potentially compromise patient safety, including preventable and non-preventable events. Adverse drug reactions (ADRs) can be defined as a subset of non-preventable adverse drug events (ADEs) in that they occur following drug administration within normal dose ranges, and they result in “noxious and unintended” consequences to the patient.^1^ ADRs are common in various healthcare settings and could lead to severe complications, increased length of stay and potential loss of life.^2^ Whilst ADRs are unavoidable, current evidence suggests that early detection of these incidents and timely intervention to manage them can significantly reduce the associated health risks.^3^,^4^ Therefore, post-marketing surveillance (eg long-term extension, Phase IV interventional or observational studies, data mining using large-scale medical databases, spontaneous reporting) remains a cornerstone of pharmacovigilance which plays a key role in providing drug safety insights.^5–7^

One of the common ADRs that many medications can cause is hyperkalemia. Hyperkalemia is defined as an electrolyte disturbance in which serum potassium level is above the upper limits of normal.^8^ While mild hyperkalemia is usually asymptomatic, high potassium levels may cause serious and potentially life-threatening implications including cardiac arrhythmias, muscle weakness, or paralysis.^9^,^10^ It is hence imperative to identify the causative factor(s) of hyperkalemia and treat it expeditiously. Drug-induced hyperkalemia is the most important cause of increased potassium levels in everyday clinical practice. A wide range of drugs can cause hyperkalemia via a variety of mechanisms.^11^ Medications can interfere with potassium homoeostasis either by impairing renal excretion of potassium, promoting transcellular potassium shift, or increasing potassium supply.^12^

Heparin and its derivates are among the most commonly used anticoagulants worldwide.^13^ They can be used for the prophylaxis and treatment of venous thromboembolism, including deep vein thrombosis (DVT) and pulmonary embolism (PE), as well as atrial fibrillation and ischemic heart disease.^14^ Heparin drugs include two types: unfractionated heparin (UFH) and low molecular weight heparin (LMWH).^14^ The widespread use of heparin has resulted in the recognition of a multitude of complications other than those due to its anticoagulant properties. This includes hyperkalemia which is presumably less well-recognized as compared to other untoward effects of heparin and possibly more common than previously thought.^15^,^16^ The proposed mechanism of heparin-induced hyperkalemia is thought to involve impaired aldosterone synthesis.^11^,^12^

Current literature provides limited epidemiologic and controlled studies on the incidence of hyperkalemia associated specifically with LMWH as compared to UFH, as well as differences in the severity of this complication and the factors that may contribute to it.^17–20^ This knowledge gap leaves clinicians without clear guidance on whether LMWH presents a comparable risk of hyperkalemia to UFH or if distinct patient factors influence severity in each case. Additionally, despite its clinical significance, heparin-induced hyperkalemia remains under-recognized by many clinicians, and there are currently no established guidelines for monitoring patients at risk. Therefore, the aim of this study is to determine and compare the incidence and magnitude of hyperkalemia, along with potential risk factors, in patients receiving UFH and LMWH in a real-life setting. By addressing these gaps, our findings may help clarify differences in hyperkalemia risk between LMWH and UFH, inform safer anticoagulant use and monitoring practices, and enhance clinician awareness of this important yet often overlooked complication.

## Methods

### Study Design and Settings

This was a retrospective observational study of all adult patients who received heparin products: UFH or LMWH (particularly dalteparin and enoxaparin) for VTE treatment or prophylaxis during hospitalization at Hamad General Hospital (HGH), Ambulatory Care Center (ACC), and Qatar Rehabilitation Institute (QRI) in Doha, Qatar. Patients were identified through a computerized pharmacy system, via an automated report generated between 1 January 2022 and 1 January 2023.

### Ethics Approval

This study was reviewed and approved by the Institutional Review Board (IRB) of the Medical Research Center (MRC) at Hamad Medical Corporation (HMC) in Qatar (approval number: MRC-01-23-342). Given that the study is retrospective, the requirement for informed consent was waived. Anonymized data were collected to maintain confidentiality, and codes were used to cover identifiers. This study was conducted in accordance with the Declaration of Helsinki.

### Study Participants

Adult patients (≥18 years) who received heparin (UFH, dalteparin, enoxaparin) during the study period and had documented potassium serum levels at baseline and during follow-up were included. Patients who received less than two doses of heparin and/or had insufficient potassium follow-up (ie less than 3 consecutive readings) were excluded from the study, as were patients with baseline hyperkalemia (before the initiation of heparin).

### Data Collection

Electronic medical records were reviewed to obtain relevant data at baseline and follow-up: socio-demographic (age, sex, ethnicity); type, dose, frequency, and duration of heparin products; indication for anticoagulation (therapeutic vs prophylaxis), comorbid conditions, and concomitant medications potentially affecting potassium serum levels including β-blockers (selective and non-selective), angiotensin-converting enzyme inhibitors (ACE-I), angiotensin receptor blockers (ARB), potassium-sparing diuretics (spironolactone, eplerenone), nonsteroidal anti-inflammatory drugs (NSAIDs), calcineurin inhibitors, digoxin, co-trimoxazole, penicillin G potassium, and antifungals (fluconazole). In addition, relevant laboratory data like serum potassium concentrations were collected. The baseline serum potassium level was defined as any level measured within one week prior to initiation of heparin, while all consecutive levels during heparin therapy (UFH or LMWH) were considered follow-up levels. “Consecutive” refers to the collective potassium levels recorded during the administration of UFH or LMWH. Hyperkalemia was defined as serum potassium level above 5.3 mmol/L.^21^ This was further categorized into mild hyperkalemia (5.3–5.9 mmol/L), moderate hyperkalemia (6.0–6.5 mmol/L) or severe hyperkalemia (above 6.5 mmol/L).

### Data Analysis

All statistical analyses were performed using the IBM SPSS (Statistical Package for the Social Sciences) version-29. Both descriptive and inferential statistics were applied for data analysis. Mean ± SD and frequency (%) were used to report on numerical and categorical variables, respectively. The level of statistical significance was defined as p≤ 0.05. Pearson’s chi-square was used to compare the incidence of hyperkalemia between different treatment groups. Multivariate logistic regression was performed to test for the association between the variables of interest and hyperkalemia. The factors included in the analysis were age, gender, indication (treatment vs prophylaxis), comorbidities [diabetes and chronic kidney disease (CKD)], and the co-administered medications. A backward elimination method was utilized to assess for the possibility of constructing a simple predictor model of hyperkalemia occurrence in the study’s cohort. Variable that resulted in a greater than 10% change in the odds of hyperkalemia were kept in the final model regardless of significance results.

## Results

This study included 929 patients who received more than two doses of any heparin product (UFH, enoxaparin, or dalteparin) over the period of 1 January 2022 and 1 January 2023. As outlined in Table 1, the majority of patients were males (n=649, 70%) with a mean age exceeding 40 years and of Arab ancestry (n=418, 45%). Hypertension was the most common comorbid disease in 40% of patients, followed by diabetes mellitus (32%) and cardiovascular disease (28%). About 50% or more of patients in each treatment cohort received at least one medication known to cause hyperkalemia; namely, beta-blockers (33%) and ACE-Is/ARBs (32%) were the most commonly co-administered medications.

**Table 1:** Participants’ Sociodemographic and Clinical Characteristics (n=929)

Variable	Heparin (n= 71)	Enoxaparin (n=515)	Dalteparin (n=343)	
**Age** mean ± SD	60.8 ± 2.2	49.1 ± 0.7	49.2 ± 0.9	<0.001^a,b^
**Male sex** n. (%)	44 (62)	364 (70.4)	241 (70.3)	0.37
**Ethnicity** n. (%)				
Arab	45 (63.3)	210 (41)	163 (47.6)	
Asian (non-Arab)	23 (32.4)	228 (44.1)	127 (37.1)	
African (non-Arab)	3 (4.2)	69 (13.4)	49 (14.3)	
Others	0	10 (2)	4 (1.2)	
**Indication** n(%)				
Prophylaxis	59 (83.1)	499 (96.5)	343 (100)	<0.001^c^
Therapeutic	12 (16.9)	18 (3.5)	0	
**Comorbidities** n. (%)				
Hypertension	40 (56.3)	219 (42.4)	113 (32.9)	<0.001^c^
Diabetes	34 (47.9)	162 (31.3)	100 (29.2)	0.01^a,b^
Cardiac Disease or ASCVD	25 (35.2)	164 (31.7)	71 (20.7)	<0.001^a,b^
Chronic Kidney Disease	19 (26.8)	11 (2.1)	11 (3.2)	<0.001^b,d^
Thyroid diseases	6 (8.5)	14 (2.7)	16 (4.7)	0.04 ^a,b^
VTE/PE/thrombus	2 (2.8)	9 (1.7)	1 (0.3)	0.09
**Medications** n. (%)				
Receiving any hyperkalemia inducing medication *	36 (50.7)	301 (58.2)	170 (49.6)	0.03^a,d^
Receiving 2 or more hyperkalemia inducing medication	19 (26.8)	152 (29.4)	85 (24.8)	
ACE-Is/ ARBs	18 (25.4)	180 (34.8)	97 (28.3)	0.07
Beta blockers	29 (40.8)	188 (36.4)	93 (27.1)	0.01^a,d^
Co-trimoxazole	4 (5.6)	32 (6.2)	14 (4.1)	0.40
NSAIDs	10 (14.1)	105 (20.3)	72 (21)	0.41
Others ^#^	4 (5.6)	14 (2.7)	11 (3.2)	

**Note**: *Receiving hyperkalemia inducing medications regardless of the number, ^#^Other medications including digoxin, azole antifungals, calcineurin inhibitors, penicillin G, potassium sparing diuretics. ^a^significant difference between heparin and enoxaparin, ^b^significant difference between heparin and dalteparin, ^c^significant difference between all groups, ^d^significant difference between enoxaparin and dalteparin.

**Abbreviations**: ACE-Is, Angiotensin-converting enzyme inhibitor; ARBs, Angiotensin 2 receptor blockers; ASCVD, Atherosclerotic cardiovascular disease; NSAIDs, Non-steroidal anti-inflammatory drugs; PE, Pulmonary embolism; VTE, Venous thromboembolism.

Enoxaparin emerged as the most frequently used anticoagulant in the study cohort (n=515, 55%), followed by dalteparin (n=343, 37%), and heparin (n=71, 8%). Table 2 highlights the distribution of hyperkalemia among study groups. Hyperkalemia was documented in 19% of study participants (n=181), with the majority of cases across all medications classified as mild (73%), along with varying proportions of moderate (15%) and high severity (12%).

**Table 2:** Frequency of Patients with Hyperkalemia Among the Three Medication Groups

Variable	Heparin	Enoxaparin	Dalteparin	P value
**Hyperkalemia** n. (%)	40 (56.3)	81 (15.7)	59 (17.2)	<0.001
**Hyperkalemia severity** n. (%)				0.18
Mild	26 (65)	57 (70.4)	49 (81.7)	
Moderate	8 (20)	14 (17.3)	5 (8.3)	
High	6 (15)	10 (12.3)	6 (10)	

**Note**: Mild hyperkalemia: potassium levels of 5.3 mmol/l - 5.9 mmol/l; Moderate hyperkalemia: potassium levels of 6 mmol/l - 6.5 mmol/l; Severe hyperkalemia: potassium levels of >6.5 mmol/l.

The incidence of hyperkalemia varies significantly depending on the anticoagulant used; specifically, 56.3% of patients receiving heparin developed hyperkalemia compared to enoxaparin (15.7%) and dalteparin (17.2%) (p<0.001), no significant difference in hyperkalemia rates was found between enoxaparin and dalteparin use (p>0.05). No statistically significant difference in hyperkalemia severity among the three medications was demonstrated (p=0.183).

A multivariate logistic regression including variables such as age, gender, diabetes, CKD, β-blockers, ACE-I/ARBs, NSAIDs, co-trimoxazole, receiving hyperkalemia-inducing medication, indication, and anticoagulant choice yielded a statistically significant model (p <0.001). After adjusting for all the variables, the use of enoxaparin (AOR 0.14, 95% CI 0.07–0.25; p <0.001) or dalteparin (AOR 0.16, 95% CI 0.08–0.29; p <0.001) was associated with significantly reduced odds of developing hyperkalemia compared to heparin. Additionally, after adjusting for other variables, diabetes mellitus (AOR 1.81, 95% CI 1.19–2.75, p<0.01) and co-trimoxazole use (AOR 2.63, 95% CI 1.34–5.13, p<0.01) were identified as predictive factors for developing hyperkalemia.

A backward elimination process was utilized to identify an appropriate model to predict the incidence of hyperkalemia in this cohort. The final variables included in the model are illustrated in Table 3. During this process, variables such as age (AOR 0.99, 95% CI 0.99–1.01), gender (AOR 0.93, 95% CI 0.64–1.37), the use of β-blockers (AOR 1.08, 95% CI 0.68–1.71), ACE-Is/ARBs (AOR 1.01, 95% CI 0.64–1.61), and NSAIDs (AOR 1.03, 95% CI 0.64–1.66) were removed from the model due to insignificant on the outcome of interest and other variables in the model (p > 0.6). Other variables that were not statistically significant but were retained in the model included CKD (AOR 1.42, 95% CI 0.65–3.10, p=0.37), concurrent use of two or more hyperkalemia-inducing agents (AOR 1.55, 95% CI 0.92–2.60, p=0.1), and the indication for anticoagulant use (AOR 0.46, 95% CI 0.17–1.30, p=0.14).

**Table 3:** Final List of Variables Proposed for the Regression Model Predicting the Incidence of Hyperkalemia

Variable	P value	AOR	95% CI
Diabetes	<0.01	1.79	1.24–2.58
Chronic kidney disease	0.37	1.42	0.65–3.10
Co-trimoxazole	0.02	2.24	1.34–4.43
Receiving 2 or more hyperkalemia inducing medication	0.10	1.55	0.92–2.60
Indication (ref. prophylaxis)	0.14	0.46	0.17–1.30
Enoxaparin (ref. heparin)	<0.001	0.14	0.08–0.26
Dalteparin (ref. heparin)	<0.001	0.16	0.09–0.30

**Abbreviations**: CI, Confidence interval; AOR, Adjusted odds ratio.

The choice of heparin product utilized remained statistically significant after adjusting for the aforementioned variables (Table 3); using enoxaparin reduced the odds of developing hyperkalemia by 86% compared to heparin (AOR 0.14, 95% CI 0.08–0.26, p<0.001), while dalteparin usage had 0.16 the odds of developing hyperkalemia compared to heparin (AOR 0.16, 95% CI 0.09–0.30, p<0.001).

## Discussion

We investigated the incidence and severity of hyperkalemia in patients receiving UFH, enoxaparin, or dalteparin, along with other potential predictors of hyperkalemia. Our findings demonstrated that the usage of UFH resulted in significantly higher incidence (56.3%) of hyperkalemia as compared to enoxaparin (15.7%) and dalteparin (17.2%) (p<0.001). In fact, the usage of enoxaparin and dalteparin significantly reduced the odds of developing hyperkalemia by 86% and 84%, respectively. Additionally, patients with diabetes mellitus or those concurrently using co-trimoxazole had significantly increased odds of developing hyperkalemia by 1.79 and 2.244, respectively. However, neither the presence of CKD nor the use of two or more hyperkalemia-inducing agents was found to be statistically significant yet they were kept in the model as they were associated with more than 10% increase in the odds of hyperkalemia.

Cases of heparin-induced hyperkalemia have been reported multiple times in the literature.^15^,^22–24^ This observed hyperkalemia can be explained by the ability of heparin to downregulate angiotensin-2 receptors on zona glomerulosa in adrenal glands, resulting in lower levels of aldosterone.^12^ Besides case reports, observational studies and clinical trials have investigated this association, with some yielding similar results to ours, while others have reported contradictory findings. In a drug monitoring study on patients taking heparin, 13 cases of hyperkalemia were documented out of 154 participants.^19^ They also reported increased frequency of hyperkalemia in patients with diabetes mellitus, which is aligned with the increased odds of hyperkalemia in patients with diabetes mellitus (OR 1.79, 95% CI 1.241–2.581, p = 0.002) reported in our study. Additionally, another study conducted by Gheno et al highlighted an increased levels of serum potassium in patients receiving LMWH (p<0.0001).^25^ The latter also found a significant association with renal insufficiency, which was insignificant in our population (p=0.374).

As our results indicated, multiple studies have suggested a benefit of LMWH over UFH. Hottelart et al compared the pre-dialysis potassium serum levels in 11 chronic hemodialysis patients taking either UFH or LMWH. They found that the mean potassium level was significantly higher in UFH group (5.66 ± 0.83) as compared to LMWH (5.15 ± 0.68) (p=0.01). Moreover, they reported significantly higher mean plasma aldosterone to plasma renin activity ratio in LMWH group as compared to UFH group (p< 0.05).^26^ Although Hottelart et al focused only on chronic hemodialysis patients, their results were consistent with ours, which indicates the applicability of this association across different populations. Moreover, Ezzatzadegan Jahromi et al reported significant reduction in potassium levels when switching hemodialysis patients from UFH to LMWH, indicating that LMWH could be a potential alternative to UFH.^27^ This is of particular importance, as it was previously believed that the use of LMWH was contraindicated in patients with deteriorating renal function due to concerns over its accumulation and potential for increased bleeding risk.^28^ Historically, UFH was preferred in these patients because of its shorter half-life and easier reversibility. However, this emerging evidence has challenged this view, suggesting that LMWH may be safely used in patients with renal impairment, provided appropriate dose adjustments are made. This shift in understanding could have significant implications for clinical practice, particularly in managing anticoagulation in patients with CKD or acute renal dysfunction.

A post-hoc analysis of the PART trial, which compared the risk of hyperkalemia in patients with coronary artery disease receiving LMWH versus placebo, found no significant difference between the two groups, suggesting no risk of hyperkalemia with certoparin (LMWH) up to 8000 I.U. aXa.^29^ Similarly, Abdel-Raheem et al detected a nonsignificant increase in serum potassium levels (p=0.09) in 28 patients taking LMWH for DVT prophylaxis following surgery.^30^ While our findings suggested a protective effect for LMWH compared to UFH, we observed significantly higher odds of hyperkalemia in patients receiving enoxaparin and dalteparin, which contrasts with these earlier studies. On the other hand, Bengalorkar et al reported a higher drug-induced increase in serum potassium levels with LMWH compared to UFH in patient with cardiovascular diseases and stroke, although the difference was deemed insignificant.^31^ These conflicting findings may stem from differences in study designs, patient populations, and clinical settings. The PART trial and Abdel-Raheem et al’s study involved specific subgroups of patients, such as those with coronary artery disease or post-surgical DVT prophylaxis, potentially limiting their generalizability. Additionally, variations in LMWH types, dosing regimens, and monitoring practices could explain the discrepancies. Differences in baseline characteristics, such as renal function and the concurrent use of medications known to affect potassium levels, could further influence outcomes. These inconsistencies highlight the need for larger, well-designed studies to better understand the risk of hyperkalemia associated with LMWH and UFH across diverse patient populations.

Our study included the largest cohort to date (n=929), which enhances its precision and generalizability of the findings. Generalizability was further strengthened by the inclusion of participants from multiple ethnic backgrounds, such as Arab, Asian, and African populations, with Arabs beings the predominant ethnicity. Additionally, we did not restrict the study to a specific patient subgroup, ensuring broader applicability. Although the majority of participants were males, which can interfere with generalizability, this distribution reflects Qatar’s population demographics. Furthermore, our study was the only one to classify the severity of hyperkalemia (mild, moderate, severe), highlighting the possibility of moderate (15%) and severe (12%) hyperkalemia. This is crucial as with the increase in severity of hyperkalemia, the risk of complications, like arrhythmias, increases.^11^ This is specifically significant in individuals with contributory factors like diabetes mellitus and CKD.^18^ For this reason, the CKD variable was retained in our final model despite its lack of statistical significance, recognizing its clinical relevance in hyperkalemia risk stratification.

The retrospective design of this study introduces inherent limitation as it relies exclusively on data collected and documented during routine clinical practice. This reliance restricts the ability to control for all potential confounding factors, leading to residual confounding. For instance, dietary potassium intake, which can vary widely among patients, was not accounted for, despite its potential to significantly influence serum potassium levels. Other clinical variables, such as hydration status, undiagnosed conditions affecting potassium regulation (eg acute kidney injury), the clinical setting (eg intensive care unit), and adherence to prescribed therapies, may have further contributed to unmeasured confounding. Another limitation of this study is the significantly smaller number of patients in the UFH group compared to the LMWH group, which may introduce bias. However, this imbalance is likely representative of clinical practice, as UFH is typically reserved for patients with altered kidney function, leading to a smaller number of patients on UFH. This aligns with our findings, which showed that patients receiving UFH were more likely to have CKD and be older, with older age being a factor that increases the risk of CKD.

To address these limitations, prospective studies with comprehensive and standardized data collection are needed. Such studies should include detailed assessments of dietary intake, medication use, and other clinical factors influencing potassium levels. We also recommend revisiting clinical guidelines on heparin use, with an emphasis on frequent monitoring of serum potassium levels to mitigate the risk of serious complications. Furthermore, we advocate for prioritizing LMWH over UFH, as it has been associated with a lower risk of potassium elevation.**5**

## Conclusion

Heparin (UFH, LMWH) administration was associated with a high risk of hyperkalemia, especially among patients with diabetes mellitus and those receiving co-trimoxazole. Whilst all heparin products led to significant increases in serum potassium levels, UFH was associated with significantly higher incidence compared to dalteparin and enoxaparin. Caution is advised when using UFH and LMWH, particularly in patients with additional risk factors that may influence serum potassium levels.

## Data Availability

No additional datasets were generated or analyzed during the current study.
